# Supplementary material for: Rock and fault rheology explain differences between on fault and distributed seismicity
Source: Nat Commun. 2022 Sep 26;13:5627. doi: 10.1038/s41467-022-33373-y (PMC9512795; doi:10.1038/s41467-022-33373-y)
Supplement: Supplementary file 1 — Supplementary Information [file 41467_2022_33373_MOESM1_ESM.pdf]

Rock and fault rheology explain differences between on fault and distributed seismicity

Cristiano Collettini et al.,

Supplementary information

Contents

Notes

Supplementary Note 1 page 2

Supplementary Note 2 page 3

Supplementary Note 3 page 4

Supplementary Note 4 page 5

Figures

Supplementary Fig. 1 page 6

Supplementary Fig. 2 page 7

Supplementary Fig. 3 page 8

Supplementary Fig. 4 page 9

Supplementary Fig. 5 page 10

Supplementary Fig. 6 page 11

Supplementary Fig. 7 page 12

Table

Supplementary Table 1 page 13

Supplementary References page 13

Notes

Note 1 Interpretation of the seismic reflection profiles

The cross section reported in Fig. 2a of the main text has been reconstructed through the interpretation of surface geology and seismic reflection profiles, starting from the well-known stratigraphy of the Umbria-Marche Apennines[1]. The overall structure consists of three major tectonic units, stacked during the Late Miocene-Early Pliocene Apennine compressional phase and involving the Jurassic-Paleogene carbonates and the underlying Triassic Evaporites, TE. From top to bottom and from west to east the major thrusts and related anticlines each forming a tectonic unit are: the Sibillini thrust, the Sarnano thrust and the Acquasanta thrust (Supplementary Fig. 1 and Supplementary Fig. 2).

In the seismic profile NOR03 the Acquasanta anticline, that is exposed at the surface, is also well imaged at depth by the seismic reflectors of the carbonates, the TE reflectors and the Acquasanta thrust (Supplementary Fig. 2). The anticline is imaged as a large asymmetric, east-verging, box-fold, with a gently west-dipping back limb and a relatively flat crestal zone[2]. In the thrust footwall, beneath the crest zone of the box fold, the seismic profile shows a set of gently E-dipping, parallel reflections, down to a depth of at least 2.5 s Two Way Time, TWT, corresponding to a deeper tectonic unit, still consisting of the Umbria-Marche sedimentary multilayer (carbonates and TE). At depth of about 3.2 s TWT, prominent reflections are associated to the top of the acoustic basement.

The NOR02 section (Supplementary Fig. 2) shows the geometry of the Sarnano anticline and its related thrust. Below the thrust, the carbonates and TE reflectors represent the prosecution towards west of the Acquasanta anticline. In the hangingwall of the thrust, the carbonates and the underlaying TE of the Sarnano anticline are present. Note that in this E-W section above the carbonates the Miocene turbidites are present in the footwall of the Sibillini thrust and they extensively crop out (Supplementary Fig. 1). In the NE-SW trending section of Fig. 2a of the main text, in the footwall of the Sibillini thrust, the top of the carbonates is present and beneath the thrust 2 km of carbonates and 2 km of TE form the back limb of the Sarnano anticline. This is the portion where clusters C2 and C3 are located within the TE. In the deeper part of the section, the top of the acoustic basement is located at about 3.2 s TWT (Supplementary Fig. 2 and [2]). The continuity of the top basement is abruptly interrupted to the west where the Sarnano thrust and the M. Vettore

normal fault merge at depth. The crosscutting relationships between these two discontinuities are ambiguous and discussed extensively in [3].

The NOR02 and NOR01 seismic profiles show a view of the high-angle normal fault systems of Norcia and Vettore (Supplementary Fig. 2). These two systems are imaged as steep alignments of disrupted reflectors that merge at the surface with mapped Quaternary normal faults[3]. The seismic volume within these conjugate faults is characterized by a seismic fabric dominated by high-angle discontinuities interpreted as a pervasive system of minor synthetic and antithetic normal faults[3]. A further, major antithetic blind normal fault can be traced in the footwall of the Norcia fault. In NOR01 in the hangingwall of the Norcia normal fault, the acoustic basement is imaged at 2.7 s TWT (Supplementary Fig. 2).

Note 2 Geometry of the activated structures

In this work we used the earthquake catalogue published in [4]. This is a machine-learning-based high-resolution earthquakes catalogue composed of ~900.000 events recorded mostly in the year after Amatrice mainshock, on 24 August 2016, from 15 August 2016 to 15 August 2017[4]. The absolute horizontal and vertical location errors have median values of 0.29 and 0.57km, respectively. In Supplementary Fig. 3 we present five cross sections oriented perpendicular to the strike of the Norcia mainshock (155°, from the TDMT solution[5]) and two sections parallel to the strike of the Norcia event. Section 3 shows the geometry of the SW-dipping fault that hosted the Norcia mainshock, and the distributed seismicity located down-dip in the hangingwall of the mainshock rupture (DHwS, cf. Fig. 2 of the main text). This DHwS shows a significant along-strike continuity since it can be followed on sections crossing the study area both in NNW (sections 1-2 in in Supplementary Fig. 3) and SSE portions (sections 4 in Supplementary Fig. 3). The geometry of this DHwS can be also appreciated along sections parallel to the strike (sections 6 and 7 in Supplementary Fig. 3). In longitudinal cross-sections, imbricated seismicity bands, that are up-to 4 km thick, are present at depths between 5-9 km. The base of the imbricated bands coincides with the top of the basement (dashed white lines) that is affected by compressional steps. These compressional steps result from thrusts rooted into the basement, formed during the Late Miocene-Early Pliocene compressional tectonic phase[1]. In our interpretation, the seismicity bands at 5-9 km of depth are hosted within Triassic Evaporites resting on top of the basement (sections 6 and 7 in Supplementary Fig. 3). SE of the Norcia mainshock, at depth of about 9-12 km a gently east-

dipping structure is present (red arrows in sections 4 and 5 of Supplementary Fig. 3). This structure, highlighted by continuous seismicity alignments, has been interpreted by previous authors as an extensional detachment[6-8].

Note 3 On fault vs. distributed seismicity: selection criteria

The details of the selected events for on fault and distributed seismicity are presented in the method section of the present work. Here we are providing the motivations for the selection criteria. For on-fault seismicity we refer to the seismicity occurring along the major active structures of the Apennines where the mainshocks nucleate. For the Norcia mainshock the earthquake fault is well-imaged by aftershock distribution from the hypocentral depth, 6.1 km, to about 2 km, and co-seismic fault slip is occurring only up-dip from the nucleation point (Supplementary Fig. 4a). Below the hypocentral depth, aftershock distribution does not show a systematic alignment that can be interpreted as the prosecution at depth of the Norcia mainshock earthquake fault (Supplementary Fig. 4a and b). In addition, at depths greater than 6 km, seismic reflection profiles do not provide a clear image for the continuity at depth of the Vettore fault and its relationship with the Sarnano thrust (see Supplementary Note 1). Although seismic reflection profiles do not show a clear picture for the normal vs. thrust faults relationship, some important considerations can be drawn from first order geological observations. It is evident from geological maps (Fig. 1, Supplementary Fig. 1 and [1]) that active normal faults have a significantly different orientation (striking NNW-SSE and dipping 30°-70°) than thrust faults (striking N-S or NNE-SSW and dipping 0°-40°). This geological observation implies that the thrusts are not optimally oriented in the regional extensional stress field and, hence, their systematic reactivation by normal faulting is extremely unlikely during the occurrence of the main shock and associated aftershocks. We interpret the zone below the hypocentral depth (depth > 6.1 km) as a zone made of structural and lithological heterogeneities within the TE, where the Norcia earthquake fault is not present. We refer to the seismicity occurring below the hypocentral depth (Supplementary Fig. 4b and c) as distributed seismicity. We define as distributed seismicity also the seismicity concentrated along the sub-vertical clusters C1-C3 in figure 2 of the main text

Note 4 Relationship between seismicity on clusters C2-C3 within TE and on the carbonates located above and below

The seismicity in the carbonates below clusters C2-C3 is not clustered in space and in time (Fig. 2 main text), but it occurs during the entire year of the catalogue and therefore there is no connection with the seismicity located on clusters C2-C3 occurring on September-October 2016 (Fig. 3g-h main text). The seismicity in carbonates above C2-C3 occurs approximately during the same time-interval of C2-C3 events. Hence, we performed further analyses on the dataset to test whether a ductile and aseismic event started in TE might have triggered seismicity on the carbonates above. To do that we have plotted in space and time the seismicity occurring in C2-C3 and in the carbonates above. All these events are highlighted in purple in Supplementary Fig. 7a and plotted in map view (Supplementary Fig. 7b) and cross-sections (Supplementary Fig. 7c-d). The grey circles (only clearly visible for M > 1.5) around the hypocentres in Supplementary Fig. 7b-d represent the rupture dimensions, estimated assuming a circular rupture and a constant stress-drop of 3 MPa e.g.,[9]. The cross sections show that most of the seismicity within the carbonates occurs in September 2016 (blue colours), whereas the seismicity within the TE mostly occurs in October with some deeper events (3-4 km) nucleating in September. Therefore, the lag observed between events in carbonates and TE suggests that a possible aseismic event starting in TE and migrating into the carbonates is unlikely. At the same time, we note an evolution in time of the seismicity within the TE volumes (see blue and yellow-red events in Supplementary Fig. 7c-d). These observations are certainly very interesting and worth further investigations.

Figures

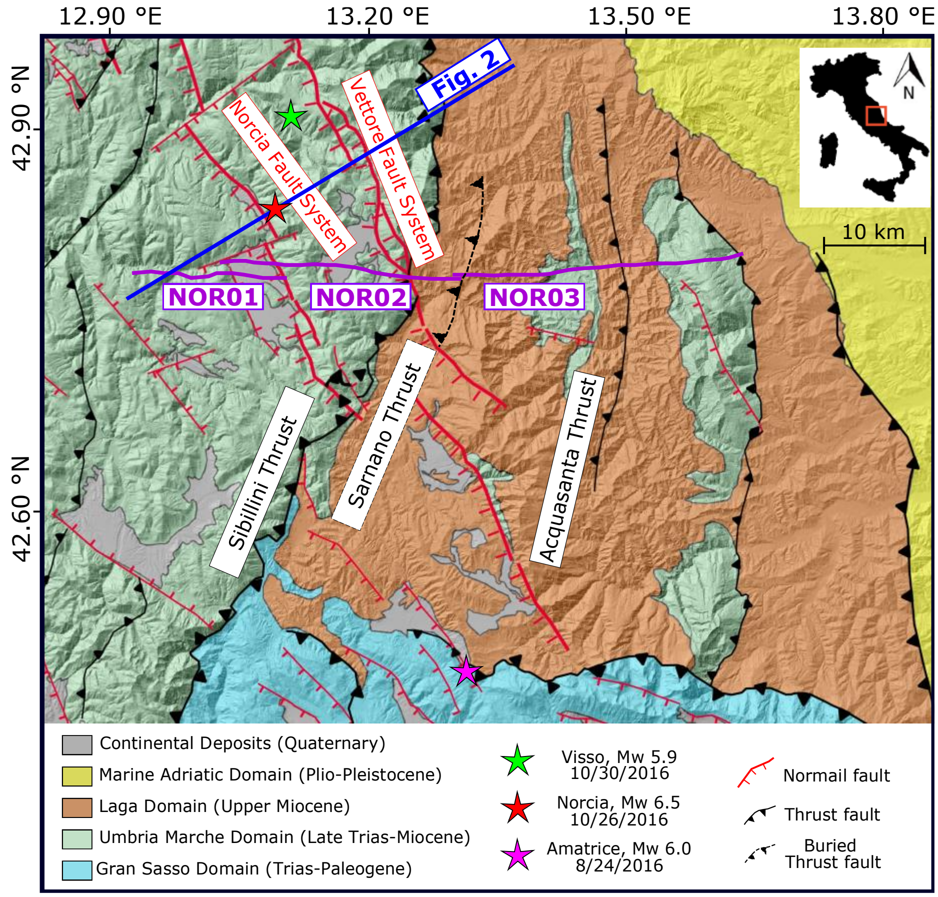

Supplementary Fig. 1. Schematic geology of the investigated area with the major extensional and compressional structures. NOR1-3 are the traces of seismic reflection profiles used to reconstruct the geological cross-section presented in figure 2 of the main text.

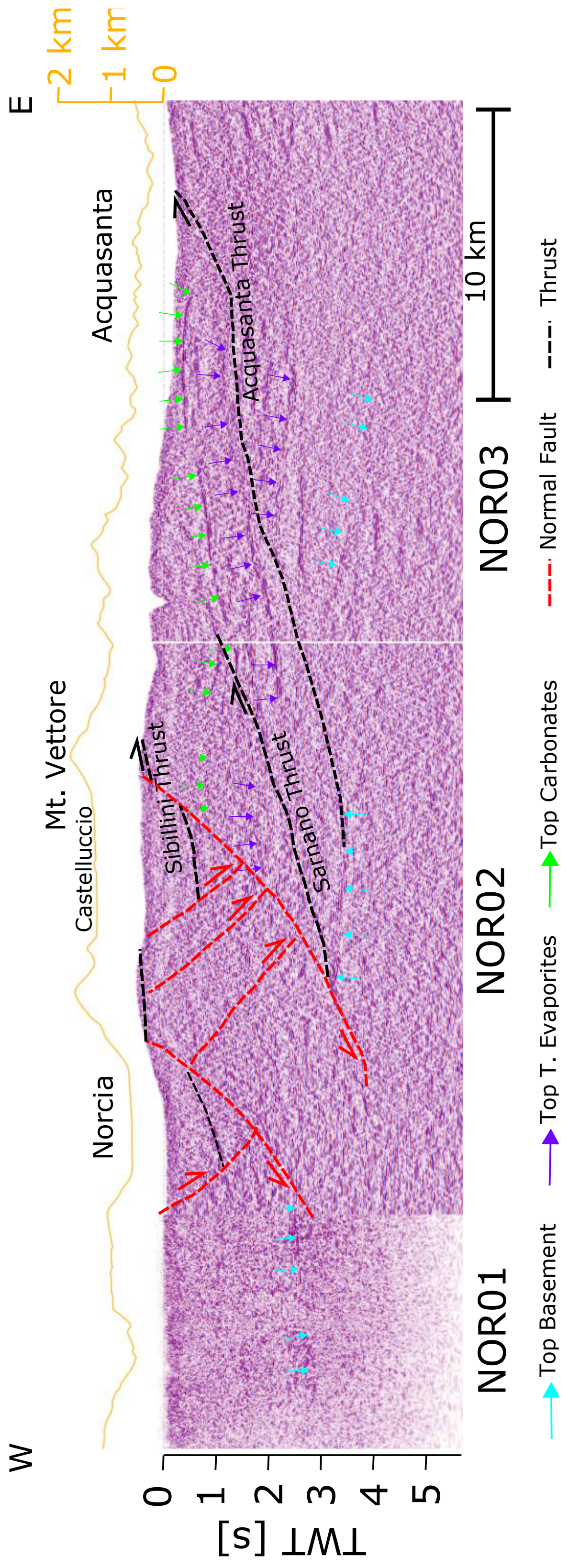

Supplementary Fig. 2. Composite seismic reflection profile merging seismic lines NOR01, NOR02, and NOR03 (cf. location in Supplementary Fig. 1) and scheme of the main reflectors.

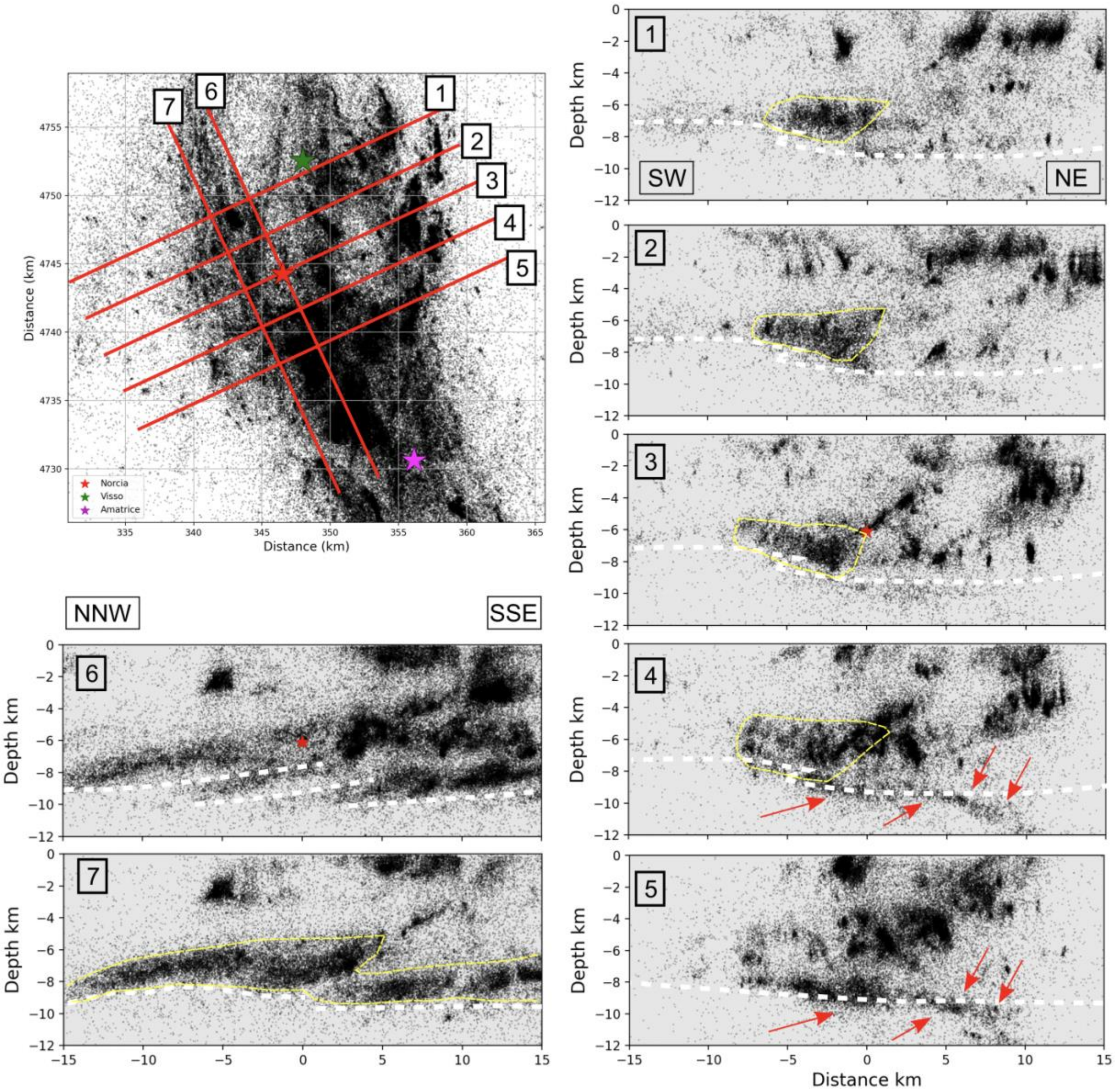

Supplementary Fig. 3. Map view and cross sections for the seismicity recorded from Amatrice mainshock to 40 days after Norcia mainshock. Earthquakes located within 2 km from the cross-section are plotted. DHwS, is highlighted with dashed yellow lines. Dashed white lines represent the depth of the acoustic basement as inferred from seismic profiles[1], red arrows highlight a gently eastward dipping structure interpreted by previous authors as an extensional shear zone or a detachment[6,7,8].

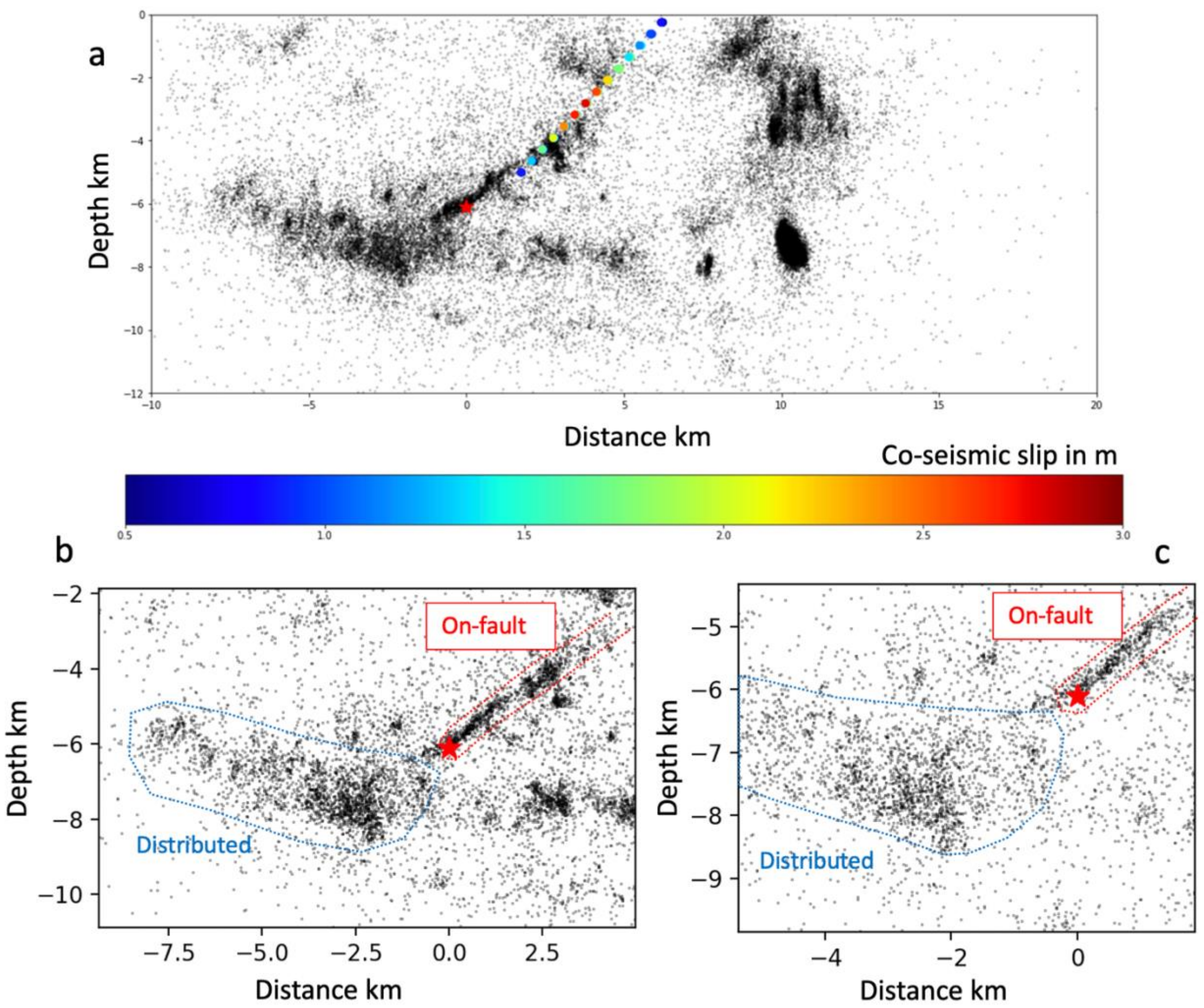

Supplementary Fig. 4. a Seismicity recorded from the Amatrice mainshock to 40 days after Norcia mainshock along a section with strike N65°, (i.e., perpendicular to the Norcia mainshock-strike[5]). The Norcia mainshock is reported in red. In all the sections we report all the seismicity located within 1 km from the cross-section. The coloured dots represent the Norcia mainshock co-seismic fault slip[5]. **b** and **c** Details of on-fault vs. distributed seismicity. For on-fault seismicity we refer to the seismicity occurring along the earthquake fault as imaged by aftershock distribution or co-seismic slip. For distributed seismicity we refer to abundant seismicity within a crustal volume where aftershock distribution or co-seismic slip do not highlight the presence of a major fault. (c) represents a detail of (b).

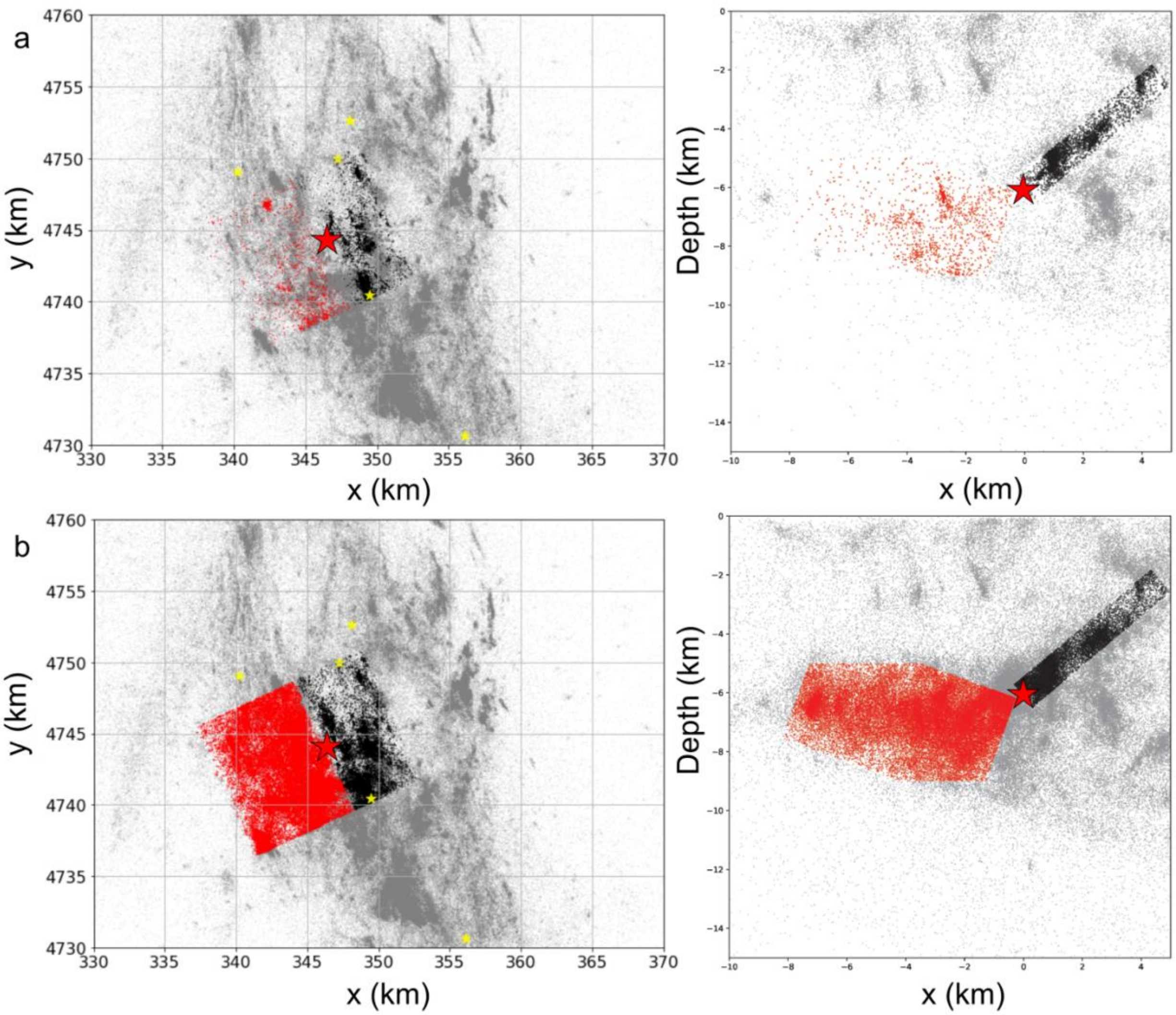

Supplementary Fig. 5. **On-fault vs. distributed seismicity. a** Seismicity before the Visso-Norcia mainshocks and (**b)** for the entire catalogue (8/15/2016-8/15/2017). Seismicity on-fault (black) and distributed down-dip in the hangingwall of the mainshock rupture, DHwS (red), plotted in map view (left) and cross section (right). The red star is the M6.5 Norcia mainshock, yellow stars in map show the events with M>5 and grey dots represent the whole catalogue.

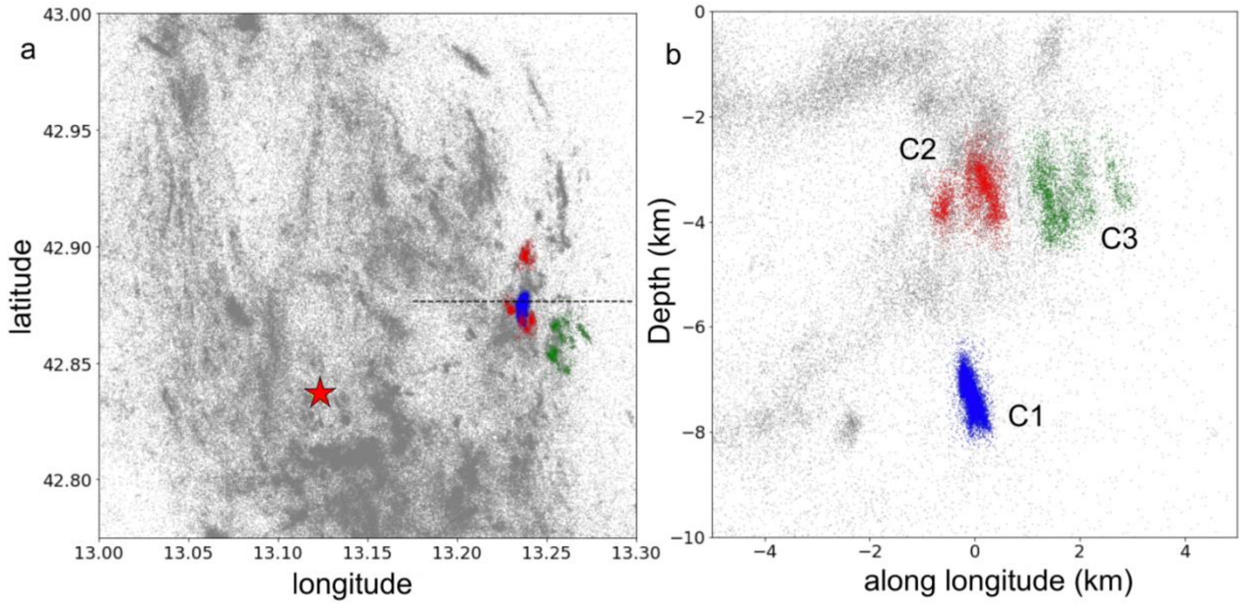

Supplementary Fig. 6. Seismicity within the selected clusters nucleating within the Triassic Evaporites. a map view and (**b**) cross section for clusters C1 in blue, C2 in red, C3 in green (see also Fig. 2). The vertical section presented in (b) is oriented along the line AA'. Red star represents the M6.5 Norcia mainshock and grey dots represent the whole catalogue.

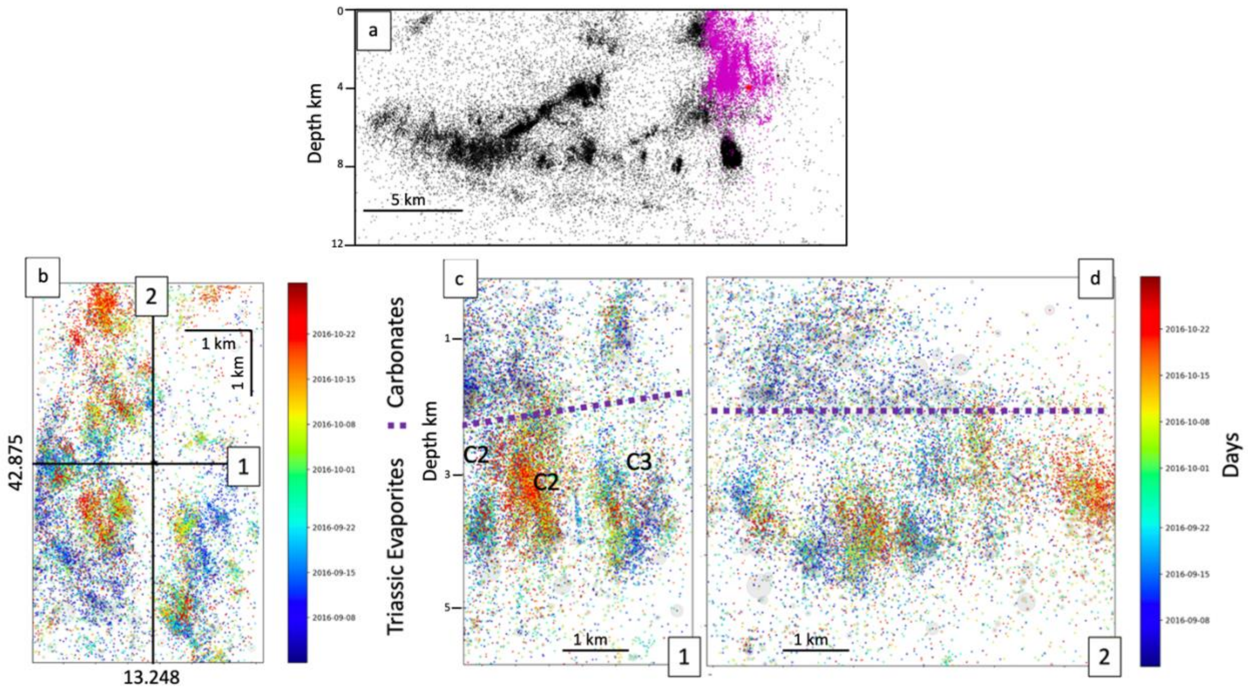

Supplementary Fig. 7. Seismicity within the carbonates located above and below clusters C2-C3. a section view of the selected (purple) seismicity along the section presented in Fig. 2 of the main text. **b** Map-view and cross-sections (**c** and **d**), for the time space evolution of the seismicity in C2-C3 and in the carbonates above. Here we plot the seismicity during the occurrence of C2-C3, i.e., before the Norcia mainshock. The grey circles around the hypocentres in b-d represent the rupture dimensions, estimated assuming a circular rupture and a constant stress-drop of 3 MPa[9].

subset	Interval of time	#events	Mc_ Lilliefors	*b*-values	95% confidence interval
On fault	Pre Visso/Norcia	3509	1.00	1.166	[1.103 1.233]
On fault	08/15/2016 08/15/2017	13379	1.00	1.344	[1.304 1.386]
Distributed DHwS	Pre Visso/Norcia	1383	1.00	1.609	[1.442 1.803]
Distributed DHwS	08/15/2016 08/15/2017	46338	1.22	1.539	[1.506 1.573]
Distributed Cluster 1	After Norcia mainshock	9383	1.06	1.807	[1. 750 1.868]
Distributed Cluster 2	Before Norcia mainshock	4144	1.00	1.714	[1.600 1.842]
Distributed Cluster 3	Before Norcia mainshock	3138	1.01	1.656	[1.541 1.785]

Supplementary Table 1. Details of the *b*-value analysis.

Supplementary references

1. Barchi, M. R., Carboni, G., Michele, M., Ercoli, M., Giorgetti, C., Porreca, M., Azzaro, S. & Chiaraluce, L. The influence of subsurface geology on the distribution of earthquakes during the 2016-–2017 Central Italy seismic sequence. *Tectonophysics* **807**, 228797 (2021).

2. Porreca, M., Minelli, G., Ercoli, M., Brobia, A., Mancinelli, P., Cruciani, F., Giorgetti, C. Carboni, C., Mirabella, F., Cavinato, G., Cannata, A., Pauselli, C. & Barchi, M. R. Seismic reflection profiles and subsurface geology of the area interested by the 2016–2017 earthquake sequence (Central Italy). *Tectonics* **37**, 1–22 (2018).

3. Ercoli, M., Forte, E., Porreca, M., Carbonell, R., Pauselli, C., Minelli, G. & Barchi, M. R. Using seismic attributes in seismotectonic research: an application to the Norcia Mw = 6.5 earthquake (30 October 2016) in central Italy. *Solid Earth* **11**, 329–348 (2020).

4. Tan, Y. J., Waldhauser, F., Ellsworth, W. L., Zhang, M., Zhu, W., Michele, M., Chiaraluce, L., Beroza, G. C. & Segou, M. Machine-Learning-Based High-Resolution Earthquake Catalog Reveals How Complex Fault Structures Were Activated during the 2016–2017 Central Italy Sequence. *The Seismic Record* **1**, 11–19 (2021).

5. Scognamiglio, L., Tinti, E., Casarotti, E., Pucci, S., Villani, F., M. Cocco et al. Complex fault geometry and rupture dynamics of the MW 6.5, 30 October 2016, central Italy earthquake. *Journal of Geophysical Research* **123**, 2943–2964 (2018).

6. Chiaraluce L. et al. The 2016 Central Italy Seismic Sequence: A First Look at the Mainshocks, Aftershocks, and Source Models. *Seism. Res. Lett.* **88**, 757-771 (2017).

7. Michele, M., Chiaraluce, L., Di Stefano, R. & Waldhauser, F. Fine-scale structure of the 2016–2017 Central Italy seismic sequence from data recorded at the Italian National Network. *Journal of Geophysical Research: Solid Earth* **125**, e2019JB018440 (2020).

8. Waldhauser, F., Michele, M., Chiaraluce, L., Di Stefano, R. & Schaff, D. P. Fault planes, fault zone structure and detachment fragmentation resolved with high- precision aftershock locations of the 2016-2017 central Italy sequence. *Geophys. Res. Lett.* **48**, e2021GL092918 (2021).

9. Kanamori, H. & Brodsky, E. E. The Physics of Earthquakes. *Rep. Prog. Phys.* **67**, (2004).
